# Supplementary material for: Association between oral microbiome and seven types of cancers in East Asian population: a two-sample Mendelian randomization analysis
Source: Front Mol Biosci. 2023 Nov 21;10:1327893. doi: 10.3389/fmolb.2023.1327893 (PMC10702768; doi:10.3389/fmolb.2023.1327893)
Supplement: Supplementary file 1 [file DataSheet1.ZIP › Supplementary Materials/MR plots for tongue/tongue═╝/Pancreatic cancer/╥╚╧┘░⌐_╔╕╤í╜ß╣√/pheno.228_to_pancreatic cancer_forest.pdf]

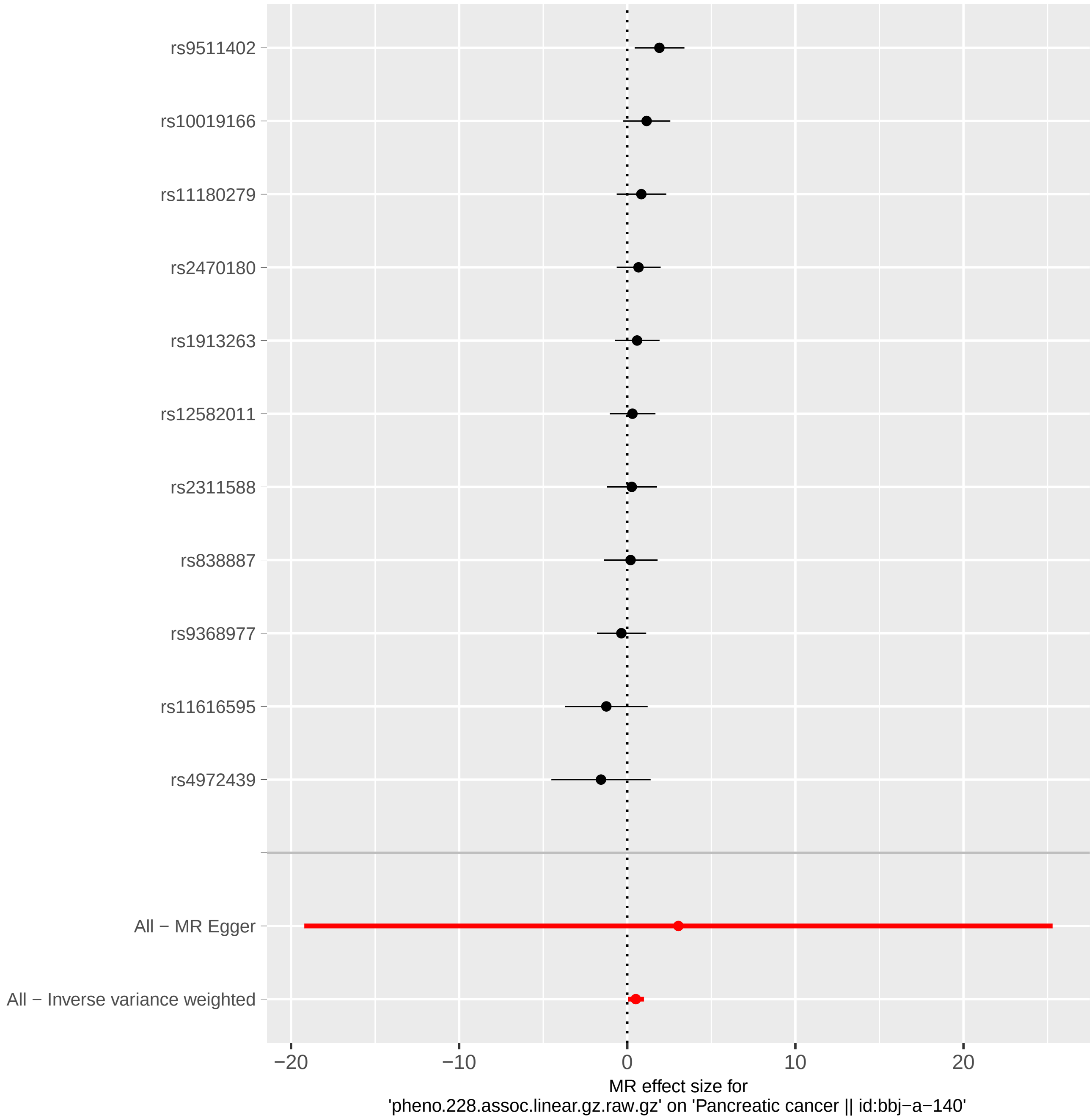
rs9511402
rs10019166
rs11180279
rs2470180
rs1913263
rs12582011
rs2311588
rs838887
rs9368977
rs11616595
rs4972439
All − MR Egger
All − Inverse variance weighted
−20
−10
0
10
20
MR effect size for
'pheno.228.assoc.linear.gz.raw.gz' on 'Pancreatic cancer || id:bbj−a−140'
